# Do budget deficits cause current account deficits? a re-evaluation utilizing military expenditures as an instrumental variable

**DOI:** 10.1371/journal.pone.0311664

**Published:** 2024-10-25

**Authors:** Haiyue Liu, Jianwu Ren

**Affiliations:** Institute of Chinese Financial Studies, Southwestern University of Finance and Economics, Chengdu, China; Flinders University, AUSTRALIA

## Abstract

The twin deficit hypothesis suggests that budget deficits are crucial factors leading to current account deficits, and substantial empirical evidence documents a significant positive statistical correlation between budget and current account deficits. However, such evidence generally suffers from endogeneity problems due to reverse causality, thereby making it difficult to provide solid evidence to support policy implementation. We use extensive cross-national panel data from developed and developing countries and military expenditure as an instrumental variable to reexamine the impact of budget deficits on the current account and test the validity of the twin deficit hypothesis. Our results demonstrate that budget deficits lead to current account deficits in both developed and developing countries, supporting the twin deficit hypothesis. After considering various factors that may affect the exogeneity of military expenditures, approximating the instrumental variable as exogenous, and performing multiple robustness tests, the hypothesis still holds. This study provides solid evidence supporting the use of fiscal policies to deal with current account imbalances.

## Introduction

Current accounts constitute a fundamental aspect of a nation’s international payments. At the level of economic practice, they influence the equilibrium of a nation’s international payments, with adjustments potentially leading to fluctuations in significant macroeconomic variables, both domestically and internationally. According to economic theory, the current account is an essential mechanism for smoothing intertemporal consumption, thus improving economic welfare and reflecting changes in domestic macroeconomic and financial stress. Consequently, maximizing the benefits of intertemporal trade through current accounts and preventing the potentially negative impacts of imbalances have long been critical issues for both theorists and policymakers. For policymakers to realize these goals more effectively, understanding the factors that determine the current account is crucial.

Among the various factors and theories explaining the determinants of the current account, the twin deficit hypothesis suggests that an increase in budget deficits leads to current account deficits or deterioration. The hypothesis affirms the impact of internal public sector imbalances on external current account imbalances, thereby suggesting significant policy implications, namely, that fiscal policy can address current account imbalances. The theoretical foundation of this hypothesis lies in the two possible channels through which fiscal policy affects the current account: intra-period and intertemporal trade. Concerning the former, the Mundell-Fleming model posits that in small open economies, an expansionary fiscal policy increases the demand for domestic goods, thus raising the domestic interest rate. Under a floating exchange rate regime, this policy would lead to capital inflows, which would increase the value of the domestic currency and thus worsen the current account. Under a fixed regime, an expansionary fiscal policy that increases domestic output also leads to an expansion in induced imports, thereby worsening the current account. Thus, under both regimes, an expansionary fiscal policy leads to a deterioration in the current account.

However, the Salter-Swan model argues that fiscal policy expansion may have two opposing effects on the current account. Expansionary fiscal spending shifts from tradable to non-tradable goods, which reduces the demand for tradable goods and results in an increase in the difference between tradable goods output and absorption, thus improving the trade balance. However, the relative increase in demand for non-tradable goods leads to real exchange rate appreciation, which causes private consumption to shift toward tradable goods and production away from tradable goods, consequently deteriorating the trade balance [1–4]. Therefore, the impact of a fiscal policy on the current account depends on the magnitude of these two effects.

In the intertemporal trade model, the influence of budget deficits on the current account is contingent on representative economic agents’ responses to their intertemporal saving and investment behaviors. In the intertemporal equilibrium model of the current account [5], a budget deficit shock is characterized as a wealth shock that merely modifies the present or future wealth of economic agents. In this framework, if economic agents are singular with indefinite lifespans, then the wealth shock is apportioned across various stages of the agent’s life, which fulfils Ricardian equivalence and thus renders the budget deficit shock inconsequential to the current account. Conversely, if agents represent overlapping generations, the wealth shock undergoes an intergenerational distribution, thereby breaching Ricardian equivalence. Consequently, the increase in the current generation’s savings is less significant than the decrease in government savings, which culminates in a current account deficit.

In the mid-1990s, the new open economy macroeconomic model (NOEM) synthesized intra-period and intertemporal models, and absorbed rational core ideas from the Mundell-Fleming and intertemporal equilibrium analysis methods. Within the NOEM, a temporary fiscal expansion results in a reduction in consumption that is less than the increase in government spending, which leads to a current account deficit. However, the effect of permanent fiscal expansion on current accounts remains ambiguous [6].

Our analysis illustrates that, aside from the Mundell-Fleming model, other theoretical frameworks have not precisely predicted the twin deficit relationship. The significance of the twin deficit hypothesis has prompted extensive empirical research on this relationship. What are the results of empirical studies? The current study summarizes the cross-national empirical evidence on budget deficits since the 1980s and reveals a significant positive correlation between budget and current account deficits, regardless of whether the former serve as explanatory or control variables. Do these empirical findings support the twin deficit hypothesis? A review and analysis of the empirical evidence reveals that previous estimates of the budget deficit coefficient suffer from endogeneity issues caused by reverse causality, which means that fiscal policy could be endogenous to the current account [7]. A country may aim for current account balance and use fiscal policy to adjust the current account. Some studies also observe or highlight this reverse causality [8–11]. Therefore, this body of empirical evidence makes it difficult to confirm the twin deficit relationship. While specifically focusing on or examining the relationship between fiscal policy and the current account, Khan and Knight [12], Debelle and Faruqee [13], Chinn and Prasad [14], Ito and Chinn [15], Bussière et al. [16], Chinn et al. [17], and Chinn and Ito [18], generally did not account for the endogeneity issue caused by reverse causality. Furthermore, some country-specific empirical evidence derived using vector autoregression (VAR) models indicates that the effect of fiscal policy on the current account differs from what the twin deficit relationship predicts [19], but owing to the small sample size, persuasive evidence is yet to be presented. Therefore, seeking instrumental variables for government budget deficits to identify the causal relationship between budget deficits and the current account is worth attempting.

This study finds that military expenditure, as an instrumental variable or proxy variable for government deficits or government spending, has been widely used in empirical research. For example, Frankel et al. [20] used military expenditures as an instrumental variable for government savings to explore the savings-investment nexus. Ramey and Shapiro [21], Burnside et al. [22], Barro and Redlick [23], and Ramey [24] employed military expenditures data to assess the influence of governmental spending on various macroeconomic variables. Hall [25] and Sheremirov and Spirovska [26] utilized such expenditures as a proxy to estimate the government expenditure multiplier. Using military expenditures as an instrumental variable for fiscal deficits, Silva [27] investigated the effects of fiscal deficits on banking credit risks and deposit loss provisions. Based on military expenditure data from 125 countries for the period 1989–2013, Miyamoto et al. [28] adopted military expenditure as an instrumental variable for government spending, and employed the local projections method to investigate the dynamic impact of government spending on the real exchange rate and the current account. Using military expenditures as an instrumental variable for budget deficits offers the following advantages. Such expenditures account for a significant part of fiscal expenditures with a high correlation to government budget deficits. Furthermore, military expenditures generally do not enter the production function and are almost unrelated to private consumption and investment [24] and thus have a minimal impact on other parts of the economy. Such expenditures are primarily influenced by international conflicts, domestic wars, arms races with neighboring countries, and military-vested interests [29,30] but are rarely associated with economic factors [25,26], thereby exhibiting strong exogeneity. Thus, military expenditure serves as an effective instrumental variable, greatly reducing the endogeneity of budget deficits, facilitating a causal analysis between budget and current account deficits, and providing robust empirical support for examining the twin deficit hypothesis and policy development.

This study aims to use military expenditure as an instrumental variable for budget deficits, employing panel data from 24 developed countries and 107 developing countries (1990–2018) to identify the causal relationship between budget deficits and the current account, thereby addressing the endogeneity problem commonly presented in previous studies. This study demonstrates that budget deficits lead to current account deficits in both developed and developing countries, supporting the twin deficit hypothesis. This empirical strategy represents the main contribution of our study relative to empirical research, thus deepening the understanding of fiscal policy and current account imbalances and providing a policy basis for ensuring sustainable economic development.

The remainder of this paper is structured as follows. The “Empirical methods and data” section details the empirical design, outlines the empirical model, empirical methods, variable metrics, and sample selection, and briefly describes the characteristics of the main variables and the exogeneity of military expenditures. The “Empirical results” section presents the empirical evidence, compares and analyzes the baseline regression results, discusses the exogeneity of the instrumental variable, and conducts various robustness tests. Finally, the “Conclusion” section offers conclusions and policy recommendations.

## Empirical methods and data

### Model setting and estimation method

We establish a reduced-form empirical model for determining the current account:

$${ca}_{i,t}=\alpha_{0}+\alpha_{1}{gov}_{i,t}+\alpha_{2}{control}_{i,t}+\mu_{i}+\delta_{t}+\varepsilon_{i,t}$$ (1)

where *ca*_*i*,*t*_ represents the current account deficit of country *i* in period *t*, *gov*_*i*,*t*_ indicates the budget deficit of country *i* in period *t*, and *control*_*i*,*t*_ represents the control variables for country *i* in period *t*. *μ*_*i*_ and *δ*_*t*_ represent country and time fixed effects, respectively, and *ε*_*i*,*t*_ is the error term.

In previous research, the coefficients of this model are frequently estimated using ordinary least squares (OLS). Owing to the reverse causality where the current account can affect the government budget deficit, the OLS estimation of the coefficient *α*_1_ for the latter is inaccurate. Hence, we adopt military expenditure as an instrumental variable for the government budget deficit and employ 2SLS for the coefficient estimation. The first-stage regression model is structured as follows:

$${gov}_{i,t}=\beta_{0}+\beta_{1}{mil}_{i,t}+\beta_{2}{control}_{i,t}+\mu_{i}+\delta_{t}+\varepsilon_{i,t}$$ (2)

where *mil*_*i*,*t*_ represents the instrumental variable for military expenditure. The control variables, *control*_*i*,*t*_, in the first-stage regression model are consistent with those in the second-stage regression model (1). We fit the first-stage empirical model to obtain the variable *gov*, which is subsequently incorporated into the second-stage empirical model for coefficient estimation, thereby yielding a more accurate estimate of the *gov* coefficient.

### Variable selection and data sourcing

#### Current account and budget deficits

The current account balance is the total trade balance of goods and services, the primary income balance, and the secondary income balance. Referring to the research [14,18,31,32], we use the current account balance as a percentage of GDP as a proxy indicator for the current account deficit, with data sourced from the IMF’s World Economic Outlook database. The government budget balance as a percentage of GDP is used to measure the extent of the budget deficit, with data sourced from the Economist Intelligence Unit database. The government budget balance in this database is total government revenue minus total expenditure. These data are sourced from national government departments, international organizations, or institutions, and their estimates cover a wide range of countries and periods.

#### Military expenditures

This study adopts the proportion of military expenditure to GDP based on data from the Stockholm International Peace Research Institute (SIPRI). Military spending in the SIPRI database includes all current expenditures on military activities and forces, such as operational costs, procurement, salaries for military personnel, and expenses on military research, development, and construction. Data in the SIPRI database are derived from various country governments, international organizations, professional journals, newspapers, and self-estimations, encompassing military expenditure data for 168 countries and regions globally.

### Control variables

(1) Economic growth rate (*gdpg*). According to the intertemporal analysis of the current account, when a country’s economy grows fastly, households will borrow foreign debt to smooth intertemporal consumption, thereby resulting in a current account deficit. Additionally, the economic growth rate reflects fluctuations in the economic cycle, and we include it in the model to control its impact on the economic cycle to some extent. Using data from the World Development Indicators (WDI) database, the economic growth rate is the real GDP growth rate.

(2) Level of economic development (*gdpp*). Similarly, when a country’s level of economic development is low, economic agents smooth consumption and borrow foreign funds over time, ultimately leading to a current account deficit. Using data from the WDI database, the level of economic development is measured by the natural logarithm of per capita real GDP adjusted by purchasing power parity.

(3) Terms of trade (*tot*). Changes in terms of trade cause changes in wealth, which leads to adjustments in the consumption and saving behaviors of economic agents and smoothing consumption over time, thus affecting the current account [33]. Based on data from Penn World Table 10.0, terms of trade are calculated as the difference between the price levels of exports and imports divided by the price level of expenditure [34].

(4) Youth dependency ratio (*yth*). In an open economy, the current account equals the gap between domestic savings and investments. On the one hand, an increasing youth dependency ratio can lead to higher household expenditures and lower savings rates [35], potentially resulting in a current account deficit. On the other hand, an increase in the ratio may also lead to a rise in household precautionary savings, thereby improving the current account balance [32]. Youth dependency ratio data are sourced from the WDI database.

(5) Old age dependency ratio (*old*). Similarly, an increase in the old age dependency ratio may either reduce the saving rate, thereby leading to a current account deficit, or increase precautionary savings, thus improving the current account. Data are sourced from the WDI database.

(6) Degree of Financial Development (*fd*). According to a viewpoint within the global savings glut hypothesis, an increase in financial development or a decrease in financial repression can diminish precautionary savings, thereby reducing the savings rate. Therefore, economies with higher degrees of financial development are more inclined to amass foreign debt, which will result in current account deficits. Using data from the WDI database, we employ the ratio of private sector credit to GDP as a proxy measure for the degree of financial development.

(7) Trade openness (*open*). Trade openness reflects the external openness policies of an economy, such as tariffs and import restrictions. These policies can affect import and export trade, thereby impacting the current account. Moreover, trade openness can influence government spending [36–38]. If the effects of trade openness are not controlled for, endogeneity issues owing to omitted variables may arise. According to data from the WDI database, this study quantifies trade openness by the share of total imports and exports in GDP.

(8) Capital account openness (*ka*). Capital account openness reflects a country’s capital openness policies. These policies can affect capital flows, thereby impacting the current account. We utilize Chinn and Ito’s [39] nominal openness index to represent the degree of capital account openness.

(9) Exchange rate regime (*errcm*). Studies document that exchange rate regimes can affect the adjustment of current account imbalances [40] as well as the strength and validity of the twin deficit relationship [10,41]. The current study includes the exchange rate regime variable in the regression to control for its impact. The regime is measured using Ilzetzki et al.’s [42] quantitative index of exchange rate regimes. The annual index used in our study is the yearly average of the monthly index.

(10) Net Foreign Assets (*nfa*). Within an open economy framework, the balance of the current account equals the sum of the trade balance and the earnings of net external assets. A greater initial net foreign asset position may lead to higher interest income for the country’s residents, thereby impacting their consumption and saving decisions and thus affecting the current account. Drawing on data from Lane and Milesi-Ferretti’s [43] External Wealth of Nations database, we adopt net foreign assets as a percentage of GDP to quantify this variable. Data on foreign net assets are available only up to 2015. Including this aspect in the regression results in a substantial loss of samples. Hence, it is incorporated into the regression only in the robustness testing section.

(11) Fiscal rules (*ER*, *RR*, *BBR*, *DR*). Badinger et al. [44] and Afonso et al. [45] established that fiscal rules can influence the strength of the twin deficit relationship. We consider four different fiscal policy rules in the regression: expenditure rules (ER), revenue rules (RR), balanced budget rules (BBR), and debt rules (DR). Data on these fiscal rules is sourced from the IMF Fiscal Rules Dataset. If a country implements a specific fiscal rule, the data for that rule are coded as 1; if a country does not implement a specific fiscal rule, they are coded as 0. Owing to severe data missing for fiscal rules in many countries, including these in the regression would result in a significant loss of sample data; hence, they are only included in the robustness checks. The definitions and sources of the variables are presented in Table 1.

**Table 1:** Variable definition and data source.

Variables	Definition and data source
*ca*	Current account balance/GDP. WDI database
*gov*	Government budget balance/GDP. EIU database
*mil*	Military expenditure/GDP. SIPRI database
*gdpg*	Real GDP growth rate. WDI database
*gdpp*	Natural log of GDP per capita adjusted for PPP. WDI database
*tot*	Difference between prices of exports and imports divided by the price level of expenditure. Penn World Table 10.0
*yth*	Population aged 0–14 years / Working-age population (15–64 years). WDI database
*old*	Population aged 65+ years / Working-age population (15–64 years). WDI database
*fd*	Private sector credit/GDP. WDI database
*ka*	The Chinn-Ito index: https://web.pdx.edu/~ito/Chinn-Ito_website.htm
*open*	Total imports and exports/GDP. WDI database
*errcm*	Quantitative Index of Exchange Rate Regimes [42].
*nfa*	Net foreign assets/GDP [43].
*ER*, *RR*, *BBR*, *DR*	Expenditure rules (ER), Revenue rules (RR), Balanced budget rules (BBR), and Debt rules (DR). IMF Fiscal Rules Dataset

### Sample selection and data processing

#### Sample selection

This study employs panel data from 1990 to 2018 for both developed and developing countries. Initially, we adopt the IMF’s classification as the standard, considering economies classified as advanced as developed countries and all other emerging markets and developing economies as developing countries. As the IMF’s classification of advanced economies changes with the number of such economies increasing over time, to ensure the stability of the country’s classification under the IMF’s dynamic standards, we use the IMF classification results from the midpoint of the sample period (2004). Additionally, the sample period begins in 1990 because many developing countries, especially the former Soviet Republics, began to gain independence or were established in the 1990s.

#### Data processing

First, based on the country-specific notes in the SIPRI database, we exclude sample data that lack comparability for reasons such as coups, civil wars, the formation and dissolution of countries, changes in military accounting systems and data sources (while retaining datasets with a longer time span that are comparable), and highly uncertain data indicated by the SIPRI database. Second, in matching data across databases, we exclude samples in which any variable data are missing for a certain period. Finally, countries without formal military forces, such as Iceland, Haiti, and Mauritius, and those like Japan, where military expenditure data fluctuate by around 1% because of defense laws or policy restrictions, are excluded. Finally, we utilize sample data from 24 developed and 107 developing countries, with relevant information about countries and years presented in S1 Table.

### Descriptive statistics

The descriptive statistics of the main variables and their correlation coefficients based on the selection of variables and samples are presented in Table 2. First, significant differences are observed between the maximum and minimum values for the current account deficit, both overall and between groups, which indicates imbalances in global current account performance. Current account deficits occur more frequently in the underdeveloped regions of Africa, Eastern Europe, and North America, while surpluses are more common in emerging Asian markets, offshore financial centers, and oil-exporting countries. Second, the within-group standard deviation of military expenditures is significantly lower than the between-group standard deviation, which suggests that military expenditures change little over time and are less likely to be influenced by common factors. From the correlation coefficients, a significant positive correlation is observed between current account and budget deficits, which is consistent with the conclusions of empirical research; however, the causal relationship of the twin deficit hypothesis requires empirical testing. Second, the current account also exhibits a significant positive correlation with military expenditure, thus indicating that military expenditure and the current account move in the same direction. Third, a significant negative correlation exists between the budget balance and military expenditures, which aligns with reality, where expanded military expenditures reduce the budget balance. Fourth, both the current account and budget deficits are significantly and positively correlated with the real GDP growth rate, which indicates that both exhibit cyclicality. Meanwhile, the correlation coefficient between military expenditure and real GDP growth rate is small and insignificant, ultimately suggesting that military spending is less affected by economic cycle fluctuations.

**Table 2:** Summary of statistics.

Variable			Mean		S.D.		Min		Max		N	
*ca*	Overall		-0.025		0.080		-0.575		0.336		2711	
	Between Groups				0.065		-0.303		0.186		131	
	Within Group				0.052		-0.514		0.333		2711	
*gov*	Overall		-0.023		0.042		-0.330		0.318		2711	
	Between Groups				0.027		-0.127		0.089		131	
	Within Group				0.033		-0.299		0.285		2711	
*mil*	Overall		0.020		0.015		0.003		0.124		2711	
	Between Groups				0.015		0.003		0.100		131	
	Within Group				0.006		-0.019		0.082		2711	
*gdpg*	Overall		0.039		0.042		-0.502		0.494		2711	
	Between Groups				0.021		-0.014		0.146		131	
	Within Group				0.038		-0.521		0.461		2711	
**Correlation coefficients between variables**												
**Coefficients**	***ρ(ca*, *gov)***	***ρ(ca*, *gdpg)***		***ρ(ca*, *mil)***		***ρ(gov*, *gdpg)***		***ρ(gov*, *mil)***		***ρ(mil*, *gdpg)***		**N**
	0.406***	-0.041**		0.007***		0.147***		-0.165***		0.014		2711

Note

*, **, and *** denote statistical significance at the 10%, 5%, and 1% levels, respectively.

### Exogeneity of military expenditures

**Fig 1:**
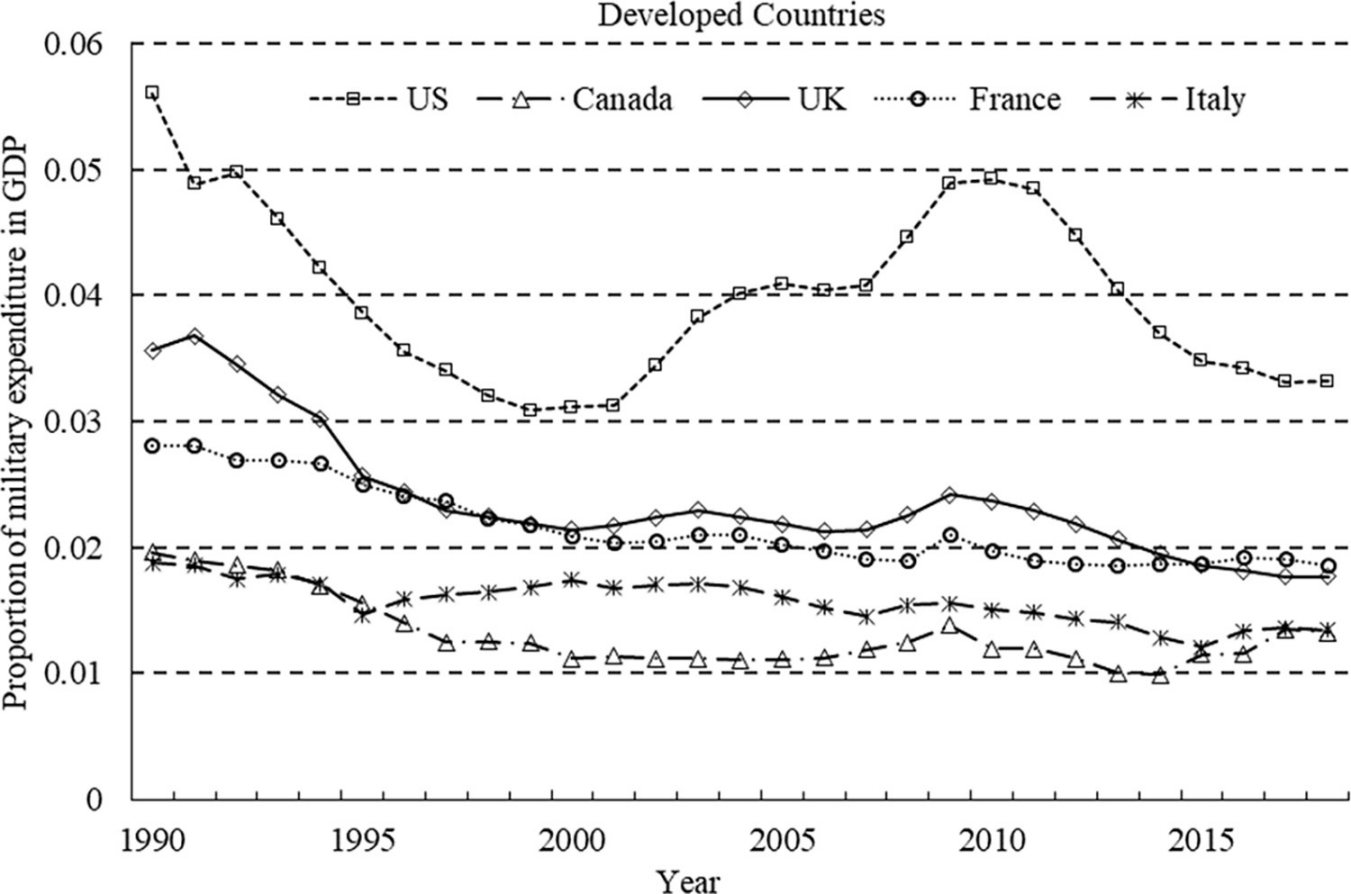
Changes in military expenditures of developed countries.

**Fig 2:**
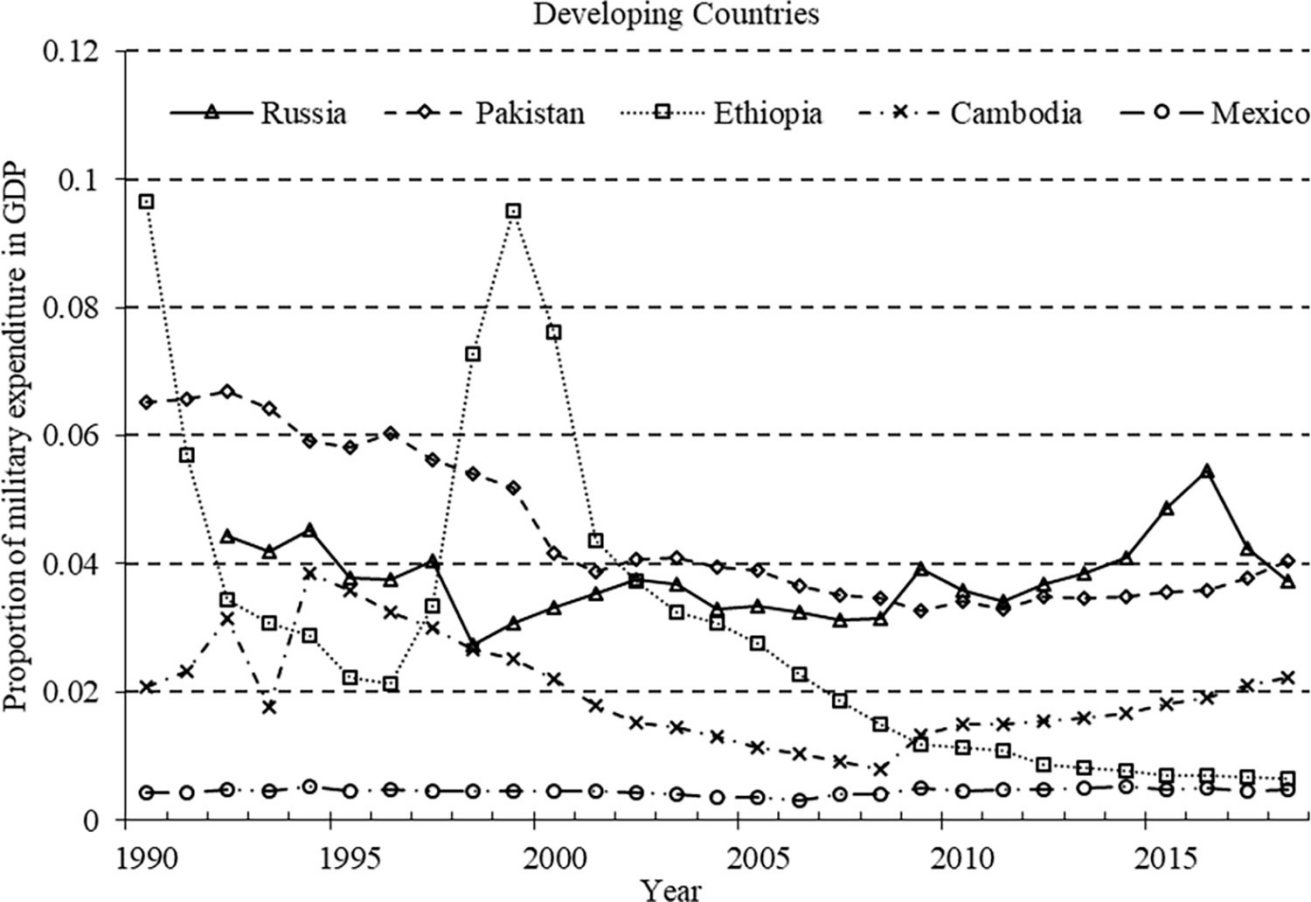
Changes in military expenditures of developing countries.

To illustrate the exogeneity of military expenditure further, we present a time series of the proportion of military expenditure to GDP in typical developed and developing countries (refer to Figs 1 and 2). In Fig 1, developed countries (as well as NATO members) witnessed a decline in the proportion of military expenditure following the end of the Cold War and the dissolution of the former Soviet Union in the 1990s. After September 11, 2001, the United States initiated wars in Afghanistan and Iraq, which led to an increase in the proportion of military expenditure. As allies, countries such as the UK and France participated in the Afghanistan and Iraq wars, and their military expenditures increased during the same period. Fluctuations in the proportion of military expenditure are relatively large in developing countries. Russia experienced an increase in the proportion of military expenditure following the two Chechen wars in 1994 and 1999 as well as after conflicts with Georgia in 2009 and Ukraine in 2014. In Africa, Ethiopia experienced a surge in the proportion of military expenditure during the border war with Eritrea from 1998 to 2000. Since the escalation of the India–Pakistan conflict in 2013, Pakistan has seen an upward trend in the proportion of military expenditure. Cambodia’s military expenditure in Southeast Asia increased after its border dispute with Thailand in 2008. In Latin America, Mexico has seen an increasing trend in the proportion of military expenditures since the launch of the drug war in 2006. These statistics document that changes in military expenditure are related to factors outside the economy, thereby demonstrating the efficacy of military expenditure as an instrumental variable.

## Empirical results

### Baseline regression results

#### Ordinary least squares estimation results

The regression findings for the impact of budget deficits on current account deficits are reported in Table 3. Columns (1)–(3) use the OLS estimation method to examine the relationship between budget and current account deficits in the full sample, developed countries, and developing countries, respectively. First, the results demonstrate that in terms of sign and significance, the regression coefficients of budget deficits are statistically significant and positive in all regressions, which is consistent with the results in numerous previous empirical studies [40,46–48]. Second, in terms of numerical value, the coefficients of budget deficits in the three regressions are 0.395, 0.312, and 0.409, respectively, which are close to those in many empirical studies [31,32,49,50]. The OLS estimation results confirm a significant positive correlation between budget and current account deficits.

**Table 3:** OLS estimation results.

	Full sample	Developed countries	Developing countries
	(1)	(2)	(3)
*gov*	0.395***	0.312**	0.409***
	(0.076)	(0.144)	(0.079)
*gdpg*	-0.130*	-0.120	-0.143**
	(0.067)	(0.136)	(0.070)
*gdpp*	0.015	-0.051	0.031
	(0.033)	(0.048)	(0.038)
*tot*	-0.004	-0.115	0.011
	(0.026)	(0.103)	(0.026)
*yth*	-0.081	0.126	-0.151***
	(0.050)	(0.207)	(0.054)
*old*	0.760***	0.113	0.719***
	(0.192)	(0.327)	(0.268)
*fd*	-0.035**	-0.021	-0.094***
	(0.014)	(0.013)	(0.022)
*open*	0.010	0.066***	0.011
	(0.015)	(0.020)	(0.018)
*ka*	-0.004	0.008	-0.004
	(0.002)	(0.006)	(0.002)
*errcm*	-0.000	0.001	-0.000
	(0.001)	(0.001)	(0.001)
Country FE	Yes	Yes	Yes
Year FE	Yes	Yes	Yes
N	2711	510	2201
R^2^	0.142	0.318	0.164

Note

*, **, and *** denote statistical significance at the 10%, 5%, and 1% levels, respectively. The values in parentheses are cluster-robust standard errors, clustered at the country level.

#### Two-stage least squares estimation results

As previously analyzed, OLS estimation faces endogeneity issues potentially caused by reverse causality, which does not clarify the causal relationship between budget and current account deficits. Therefore, we employ military expenditure as an instrumental variable for budget deficits to perform a 2SLS estimation, with the specific estimation results presented in Table 4.

**Table 4:** 2SLS estimation results.

	Full sample	Developed countries	Developing countries
	(1)	(2)	(3)
*gov*	0.815***	0.488***	0.822***
	(0.218)	(0.170)	(0.255)
*gdpg*	-0.172***	-0.165	-0.174***
	(0.057)	(0.107)	(0.058)
*gdpp*	-0.000	-0.071**	0.019
	(0.015)	(0.032)	(0.017)
*tot*	-0.003	-0.150*	0.011
	(0.018)	(0.080)	(0.018)
*yth*	-0.080***	0.113	-0.135***
	(0.025)	(0.099)	(0.031)
*old*	0.676***	0.140	0.601***
	(0.091)	(0.143)	(0.138)
*fd*	-0.029***	-0.020***	-0.078***
	(0.008)	(0.007)	(0.019)
*open*	0.003	0.069***	0.003
	(0.010)	(0.017)	(0.012)
*ka*	-0.002	0.008**	-0.002
	(0.002)	(0.004)	(0.002)
*errcm*	0.000	0.001	0.000
	(0.001)	(0.001)	(0.001)
Country FE	Yes	Yes	Yes
Year FE	Yes	Yes	Yes
N	2711	510	2201
R^2^	0.082	0.298	0.114
**First-stage regression results**			
*mil*	-1.273***	-3.007***	-1.116***
	(0.158)	(0.425)	(0.160)
K-P rk LM	50.992***	42.109***	37.608***
C-D Wald F	127.235	40.025	91.766
K-P Wald rk F	64.754	50.162	48.903
A-R Wald test F	11.77***	6.21**	8.66***

Note

*, **, and *** denote statistical significance at the 10%, 5%, and 1% levels, respectively. The values in parentheses are cluster-robust standard errors, clustered at the country level.

First, the first-stage regression outcomes indicate that military expenditure coefficients are significantly negative, thus aligning with the previously mentioned correlation analysis findings. This result suggests that an increase in military expenditures diminishes the budget balance. Furthermore, the Kleibergen-Paap rk LM statistic significantly rejects the null hypothesis that the “instrumental variable is not related to the endogenous variable” at the 1% level, which indicates the absence of a weak instrumental variable issue. The Cragg-Donald Wald F statistics far exceed Stock-Yogo’s 10% critical threshold of 16.38, and the Kleibergen-Paap rk Wald F statistics exceed 10, which suggests a lack of weak instrumental variable concerns empirically; the Anderson-Rubin Wald test F statistics are significant at the 10% level, indicating no concerns regarding weak instrumental variables.

Second, in the second-stage regression results, the regression coefficients of budget deficits are significantly positive in all regressions, which reveals that an increase in budget deficits leads to an increase in current account deficits, thus supporting the twin deficit hypothesis. Regarding numerical values, the coefficients of budget deficits in the 2SLS regressions are 0.815, 0.488, and 0.822, respectively. Economically, this result implies that holding other factors constant, on average, a 1% increase in the ratio of budget deficits to GDP leads to an increase in the ratio of current account deficits to GDP of 0.815%, 0.488%, and 0.822% in the overall sample, developed countries, and developing countries, respectively. Additionally, the twin deficit relationship is stronger in developing countries compared to in developed countries.

Finally, the coefficients of budget deficits in the 2SLS regressions are significantly larger than those in the corresponding OLS regressions, particularly in the context of developing countries, where the 2SLS coefficients exceed those in the OLS by more than double. This pronounced variance may stem from endogeneity concerns within the OLS regressions, potentially induced by reverse causality. The current account targeting hypothesis posits that governments may deploy fiscal strategies to steer the current account toward equilibrium. This strategy may entail reducing budget deficits amid current account shortfalls and augmenting them during surpluses to align savings with investments in an open economy. Under these circumstances, the OLS regressions may exhibit potential inverse causality depending on the independent variables, ultimately inducing a negative correlation between the independent variables and error terms, which results in underestimated coefficients within the OLS estimates. This analysis suggests that the higher coefficients for budget deficits derived from the 2SLS regressions than those from the OLS regressions are justified. This demonstrates that incorporating instrumental variables significantly solves the endogeneity issues in the OLS estimates, thereby adjusting the systematically underestimated coefficients of budget deficits.

Furthermore, the coefficient of *gdpg* is significantly negative in both the full and developing country samples, but insignificant in the developed country sample. This indicates that rapid economic growth leads to a current account deficit in the overall and developing country samples, while in the developed country sample, economic growth does not significantly impact the current account. The coefficient of *gdpp* is insignificant in the full and developing country samples, but significantly negative in the developed country sample, suggesting that economic development does not significantly impact the current account in the former samples, whereas in the latter sample, higher economic development worsens the current account. The coefficient of *tot* is insignificant in the full and developing country samples, but significantly negative in the developed country sample. This finding indicates that terms of trade do not significantly affect the current account in the former samples, while an improvement worsens the current account in the latter sample. The coefficient of *yth* is significantly negative in both the full and developing country samples, but insignificant in the developed country sample. This finding suggests that an increase in the youth dependency ratio worsens the current account in the former samples, while the ratio does not significantly impact the current account in the latter sample. The coefficient of *old* is significantly positive in the full and developing country samples, but insignificant in the developed country sample. This indicates that an increase in the older people dependency ratio improves the current account in the former samples, while the ratio does not significantly impact the current account in the latter sample. The coefficient of *fd* is significantly negative in the full, developed country, and developing country samples, which indicates that higher levels of financial development worsen the current account in the overall, developed country, and developing country samples. The coefficients of *open* and *ka* are insignificant in the full and developing country samples, but significantly positive in the developed country sample. This suggests that trade and capital account openness do not significantly impact the current account in the former samples, while in the latter sample, higher trade openness and capital account openness improve the current account. The coefficient of *errcm* is insignificant in the full, developed country, and developing country sample, thus indicating that the exchange rate regime does not significantly impact the current account in the samples.

### Further discussion on the instrumental variable

When using instrumental variables, two conditions must be satisfied: First, the instrumental variable must be correlated with the endogenous explanatory variable, and second, it must not be correlated with the error term. The weak instrumental variable test in the first-stage regression indicates that military expenditure satisfies the first condition. For the second condition, econometrics cannot test the exogeneity of the instrumental variable in the case of identification. However, factors such as financial crises, severe internal disturbances, terrorist activities, and weapons imports may affect the exogeneity of military expenditures. Nonetheless, apart from these factors, military spending may also directly affect people’s expectations, thus influencing the current account or indirectly impacting the current account through unobservable channels such as energy demand, the national security environment, and security advantages, not fully meeting the exogeneity requirement. Therefore, we first consider the impact of factors such as financial crises, severe internal disturbances, terrorist activities, and weapons imports and then use Conley et al.’s [51] proposed method to test the impact of the instrumental variable’s near exogeneity on the results.

#### Eliminating the influences of financial crises, significant civil unrest, terrorism, and arms imports

(1) Excluding financial crisis samples. A country experiencing a financial crisis may resort to fiscal policies for intervention. In particular, severe financial crises can exert pressure on military expenditures. A financial crisis may also cause changes to the current account, which affects the exogeneity of the instrumental variable. Following Gourinchas and Obstfeld [52], we categorize financial crises as currency, banking, and sovereign debt crises. First, a country is considered to have experienced a currency crisis if its currency depreciates more than 15% against the US dollar [53]. Second, a banking crisis is defined as a severe financial disturbance within the banking system (characterized by significant bank runs, massive losses within the banking system, or liquidation of banks) or the government taking evident intervention measures in response to significant losses in the banking system [54]. Finally, following Reinhart and Rogoff [53], a country is considered to have experienced a sovereign debt crisis if it defaults on its obligations. Based on these definitions, we exclude samples from individual countries during financial crises, leading to regressions (1)–(3) in Table 5. As the data on banking crises are only updated to 2017, the sample period for these regressions is 1990–2017.

**Table 5:** Eliminating the influences of financial crises.

	Full sample	Developed countries	Developing countries	Full sample	Developed countries	Developing countries
	(1)	(2)	(3)	(4)	(5)	(6)
*gov*	0.697***	0.467**	0.637**	0.646*	0.609	0.824*
	(0.232)	(0.238)	(0.277)	(0.334)	(0.403)	(0.485)
*gdpg*	-0.147	-0.016	-0.168	0.039	-0.250	0.117
	(0.110)	(0.120)	(0.124)	(0.143)	(0.222)	(0.168)
*gdpp*	0.047*	-0.023	0.069**	0.097*	0.022	0.143*
	(0.027)	(0.037)	(0.034)	(0.058)	(0.080)	(0.078)
*tot*	0.052	-0.221**	0.069*	0.063	-0.336***	0.091*
	(0.036)	(0.108)	(0.040)	(0.048)	(0.121)	(0.055)
*yth*	-0.019	0.182	-0.050	-0.136	0.202	-0.214
	(0.050)	(0.116)	(0.099)	(0.102)	(0.285)	(0.180)
*old*	0.537***	0.432**	0.386	-0.269	-0.154	-0.278
	(0.137)	(0.172)	(0.272)	(0.454)	(0.291)	(1.417)
*fd*	-0.056***	-0.030***	-0.104***	-0.071***	-0.043***	-0.196***
	(0.010)	(0.008)	(0.029)	(0.016)	(0.010)	(0.056)
*open*	0.020	0.051***	0.023	0.016	0.117***	-0.010
	(0.014)	(0.017)	(0.019)	(0.029)	(0.035)	(0.044)
*ka*	-0.002	0.007*	-0.002	-0.005	0.003	-0.002
	(0.003)	(0.004)	(0.004)	(0.005)	(0.006)	(0.006)
*errcm*	-0.001	0.001	-0.004**	0.001	0.003**	-0.001
	(0.001)	(0.001)	(0.001)	(0.002)	(0.001)	(0.003)
Country FE	Yes	Yes	Yes	Yes	Yes	Yes
Year FE	Yes	Yes	Yes	Yes	Yes	Yes
N	1020	393	627	540	243	297
R^2^	0.193	0.335	0.256	0.267	0.471	0.355
**First-stage regression results**						
*mil*	-1.802***	-2.710***	-1.629***	-2.014***	-1.857***	-1.695***
	(0.328)	(0.481)	(0.361)	(0.383)	(0.527)	(0.457)
K-P rk LM	25.933***	29.558***	18.838***	22.859***	10.506***	13.489***
C-D Wald F	103.772	34.997	62.027	55.638	13.374	26.287
K-P Wald rk F	30.251	31.768	20.404	27.640	12.419	13.744
A-R Wald test F	6.51**	2.80*	3.93**	3.61*	1.69	2.90*

Note

*, **, and *** denote statistical significance at the 10%, 5%, and 1% levels, respectively. The values in parentheses are cluster-robust standard errors, clustered at the country level.

Moreover, many countries’ current accounts underwent structural adjustments after the global financial crisis, and exhibited trends different from those before the crisis. Therefore, we further consider the impact of the global financial crisis by excluding samples after 2007, leading to regressions (4) and (6) in Table 5. This result indicates that even considering the potential endogeneity of the instrumental variable caused by the financial crisis, the twin deficit hypothesis holds. It should be noted, however, that excluding the impact of the financial crisis significantly reduces the sample size in the regressions, which may lead to a certain degree of weak instrument problem.

(2) Excluding samples during severe internal disturbances. Severe internal disturbances can cause significant casualties, destruction of resources and infrastructure, and population displacement, influencing social groups’ psychology and culture, thereby affecting the current account balance. Moreover, such disturbances are associated with increased military expenditure. Hence, they may affect military expenditure exogeneity. To mitigate this effect, we exclude all samples during internal disturbances based on the Major Episodes of Political Violence (MEPV) database released by the Center for Systemic Peace, resulting in regressions (1)–(3) in Table 6. The MEPV database has recorded major political violence events since 1946, involving at least 500 deaths and an average of 100 deaths per year, including international wars and conflicts, domestic internal wars and conflicts, and ethnic wars and conflicts, with the majority and the most severe being domestic turmoil. The regression results also indicate that the coefficient of the budget deficit is significantly positive, thus supporting the twin deficit hypothesis.

**Table 6:** Excluding samples during severe internal disturbances and eliminating the impact of terrorism.

	Full sample	Developed countries	Developing countries	Full sample	Developed countries	Developing countries
	(1)	(2)	(3)	(4)	(5)	(6)
*gov*	0.984***	0.417*	1.012***	0.806***	0.523***	0.819***
	(0.244)	(0.215)	(0.286)	(0.219)	(0.171)	(0.259)
*gdpg*	-0.159**	-0.156	-0.147*	-0.171***	-0.176	-0.174***
	(0.074)	(0.114)	(0.075)	(0.057)	(0.112)	(0.058)
*gdpp*	-0.009	-0.058	0.010	-0.001	-0.069**	0.019
	(0.016)	(0.036)	(0.018)	(0.015)	(0.032)	(0.017)
*tot*	0.021	-0.120	0.032	-0.003	-0.131	0.011
	(0.019)	(0.085)	(0.020)	(0.018)	(0.081)	(0.018)
*yth*	-0.052*	0.107	-0.102**	-0.078***	0.135	-0.135***
	(0.030)	(0.103)	(0.040)	(0.025)	(0.100)	(0.031)
*old*	0.613***	0.120	0.538***	0.667***	0.139	0.598***
	(0.110)	(0.146)	(0.177)	(0.091)	(0.143)	(0.137)
*fd*	-0.023***	-0.019***	-0.069***	-0.030***	-0.019***	-0.078***
	(0.009)	(0.007)	(0.024)	(0.008)	(0.007)	(0.019)
*open*	-0.001	0.069***	-0.002	0.003	0.072***	0.003
	(0.012)	(0.018)	(0.014)	(0.010)	(0.018)	(0.012)
*ka*	0.000	0.007*	0.000	-0.002	0.008**	-0.002
	(0.002)	(0.004)	(0.002)	(0.002)	(0.004)	(0.002)
*errcm*	0.001	0.001	0.001	0.000	0.001	0.000
	(0.001)	(0.001)	(0.001)	(0.001)	(0.001)	(0.001)
*terr*				-0.001	-0.006***	-0.000
				(0.001)	(0.002)	(0.001)
Country FE	Yes	Yes	Yes	Yes	Yes	Yes
Year FE	Yes	Yes	Yes	Yes	Yes	Yes
N	2300	479	1821	2711	510	2201
R^2^	0.013	0.312	0.049	0.085	0.307	0.114
**First-stage regression results**						
*mil*	-1.470***	-3.264***	-1.297***	-1.263***	-3.001***	-1.094***
	(0.214)	(0.604)	(0.216)	(0.158)	(0.428)	(0.159)
K-P rk LM	37.866***	32.784***	27.950***	50.642***	41.356***	36.959***
C-D Wald F	107.203	30.191	77.653	124.608	39.616	87.719
K-P Wald rk F	47.158	29.168	36.071	63.562	49.137	47.159
A-R Wald test F	12.71***	2.79*	9.83***	11.47***	7.13***	8.38***

Note

*, **, and *** denote statistical significance at the 10%, 5%, and 1% levels, respectively. The values in parentheses are cluster-robust standard errors, clustered at the country level.

(3) Discarding the impact of terrorist activities. Terrorist activities directly affect foreign trade [55,56], potentially influencing current accounts. Furthermore, terrorist activities and military expenditures may have a causal relationship. To combat terrorism, a country may increase its military spending, while an increase in military expenditures can enhance a country’s military strength and law enforcement capabilities, thereby suppressing terrorist activities. Considering the potential impact of this possibility on our research conclusions, we add a variable for terrorist activities (*terr*) to the model, resulting in regressions (4) to (6) in Table 6. This variable is represented by the logarithmic value of the number of terrorist incidents occurring in each country per year, where numbers less than or equal to 1 are directly considered 0, and those greater than 1 are logged. Data on terrorist activities are sourced from the Global Terrorism Database, published by the START team at the University of Maryland. The regression results indicate that the budget deficit coefficient is significantly positive, thus supporting the twin deficit hypothesis.

(4) Excluding samples from major arms-importing countries. A portion of military expenditure may be allocated to the import of weapons and equipment that are included in a country’s balance of payments. High levels of arms imports can significantly reduce trade balances, thus affecting the current account balance. This study calculates the average ratio of arms imports to military expenditures for each country from 1995 to 2017 based on the World Military Expenditures and Arms Transfers (WMEAT) database. The results indicate that the minimum, maximum, and average value of this ratio for countries worldwide is 0, less than 0.5, and approximately 0.1, respectively. Additionally, the WMEAT database indicates that the ratio of annual arms imports to total goods and services imports worldwide was less than 1% from 1995 to 2017. This result suggests that arms imports do not significantly impact military expenditures or the current account. Nevertheless, they may still affect the exogeneity of our instrumental variable, thus affecting the research conclusions. Hence, this study excludes countries where the average ratio of arms imports to military expenditure from 1995 to 2017 exceeds 25%, resulting in regressions (1)–(3) in Table 7. The regression results indicate that the budget deficit coefficient is significantly positive, thus supporting the twin deficit hypothesis.

**Table 7:** Excluding samples from major arms-importing countries.

	Full sample	Developed countries	Developing countries
	(1)	(2)	(3)
*gov*	0.716**	0.400**	0.761**
	(0.288)	(0.175)	(0.362)
*gdpg*	-0.163**	-0.179	-0.177***
	(0.063)	(0.134)	(0.065)
*gdpp*	0.011	-0.017	0.028
	(0.019)	(0.042)	(0.022)
*tot*	0.003	-0.088	0.016
	(0.022)	(0.114)	(0.023)
*yth*	-0.037	0.326**	-0.096**
	(0.029)	(0.135)	(0.039)
*old*	0.790***	0.563***	0.655***
	(0.108)	(0.160)	(0.167)
*fd*	-0.040***	-0.029***	-0.090***
	(0.010)	(0.008)	(0.025)
*open*	-0.001	0.047**	0.000
	(0.013)	(0.018)	(0.016)
*ka*	-0.003	0.012**	-0.003
	(0.002)	(0.005)	(0.002)
*errcm*	0.000	0.000	-0.000
	(0.001)	(0.001)	(0.001)
Country FE	Yes	Yes	Yes
Year FE	Yes	Yes	Yes
N	2198	393	1805
R^2^	0.138	0.379	0.150
**First-stage regression results**			
*mil*	-1.304***	-3.161***	-1.070***
	(0.209)	(0.485)	(0.217)
K-P rk LM	30.481***	34.272***	19.507***
C-D Wald F	90.474	47.842	55.238
K-P Wald rk F	38.812	42.554	24.228
A-R Wald test F	4.81**	3.96**	3.39*

Note

*, **, and *** denote statistical significance at the 10%, 5%, and 1% levels, respectively. The values in parentheses are cluster-robust standard errors, clustered at the country level.

#### Influence of approximate exogeneity of the instrumental variable

Although we exclude the impacts of various factors, military expenditure may still affect the current account directly or via other unobserved pathways, thus not fully satisfying the exclusion restriction. We further discuss two additional aspects to assess the extent of the exclusion restriction on military expenditures and verify the robustness of the findings in scenarios where such expenditures are approximately exogenous.

First, we demonstrate the exclusion restriction on military expenditure using an empirical testing approach. For this purpose, we conduct auxiliary regressions for both the full sample and subsamples (Table 8). The regression results of incorporating military expenditure as a proxy variable for budget deficits in Model (1) are reported in Columns (1)–(3), and the results of including both military expenditure and budget deficits in Model (1) in Columns (4)–(6). The results in Columns (1)–(3) indicate that the coefficient of military spending is significantly negative at the 10% level. This coefficient reflects the total effect of military expenditures on the current account, including the impact of military spending in the current account through budget deficits and potential effects through direct or other unobserved channels. The results in Columns (4)–(6) reveal that after including both variables in the model, the coefficient of budget deficits remains significantly positive at the 10% level, but that of military expenditure is no longer statistically different from 0. This result suggests that within the framework of our model setting, military expenditures affect the current account only through budget deficits rather than directly or through other unobserved channels.

**Table 8:** Auxiliary regressions.

	Full sample	Developed countries	Developing countries	Full sample	Developed countries	Developing countries
	(1)	(2)	(3)	(4)	(5)	(6)
*gov*				0.374***	0.296*	0.391***
				(0.072)	(0.147)	(0.076)
*mil*	-1.038**	-1.468**	-0.918**	-0.561	-0.578	-0.482
	(0.434)	(0.696)	(0.449)	(0.399)	(0.545)	(0.414)
*gdpg*	-0.099	-0.060	-0.119	-0.132**	-0.123	-0.145**
	(0.072)	(0.135)	(0.076)	(0.066)	(0.137)	(0.069)
*gdpp*	0.027	-0.025	0.042	0.015	-0.052	0.031
	(0.034)	(0.071)	(0.039)	(0.034)	(0.048)	(0.039)
*tot*	-0.011	-0.077	0.005	-0.007	-0.121	0.008
	(0.027)	(0.121)	(0.027)	(0.025)	(0.103)	(0.026)
*yth*	-0.076	0.182	-0.157***	-0.078	0.140	-0.147**
	(0.054)	(0.254)	(0.056)	(0.052)	(0.210)	(0.056)
*old*	0.817***	0.159	0.820***	0.752***	0.147	0.716***
	(0.207)	(0.359)	(0.275)	(0.192)	(0.318)	(0.270)
*fd*	-0.042***	-0.023	-0.110***	-0.036***	-0.021	-0.095***
	(0.015)	(0.014)	(0.023)	(0.014)	(0.013)	(0.022)
*open*	0.018	0.065**	0.021	0.011	0.067***	0.013
	(0.015)	(0.025)	(0.017)	(0.015)	(0.020)	(0.018)
*ka*	-0.005**	0.009	-0.005**	-0.004	0.008	-0.004
	(0.002)	(0.007)	(0.003)	(0.002)	(0.006)	(0.002)
*errcm*	-0.001	0.000	-0.001	-0.000	0.001	-0.001
	(0.001)	(0.001)	(0.001)	(0.001)	(0.001)	(0.001)
Country FE	Yes	Yes	Yes	Yes	Yes	Yes
Year FE	Yes	Yes	Yes	Yes	Yes	Yes
N	2711	510	2201	2711	510	2201
R^2^	0.099	0.268	0.123	0.145	0.320	0.166

Note

*, **, and *** denote statistical significance at the 10%, 5%, and 1% levels, respectively. The values in parentheses are cluster-robust standard errors, clustered at the country level.

Second, we employ the method proposed by Conley et al. [51] to test the robustness of the estimation results when the instrumental variables do not fully satisfy the exclusion restriction. The model setup for the test is as follows:

$${ca}_{i,t}=\alpha_{0}+\alpha_{1}{gov}_{i,t}+\gamma {mil}_{i,t}+\alpha_{2}{control}_{i,t}+\mu_{i}+\delta_{t}+\varepsilon_{i,t}$$ (3)

Under the exclusion restriction of instrumental variables, the value of *γ* in Model (3) should be 0, while it is non-zero when the exclusion restriction is violated. This study employs the union of confidence intervals (UCI) approach [51]. By defining the support set of *γ* as a bounded closed set, we construct an effective confidence interval for *α*_1_ for any selected *γ* within the support set. The combination of confidence intervals for *α*_1_ is obtained for all possible *γ* values, where the upper and lower limits of the combined confidence interval for *α*_1_ do not include 0, thereby indicating that the instrumental variable estimation of *α*_1_ remains significantly different from 0. Consequently, we can determine the range of possible values for *γ*.

**Fig 3:**
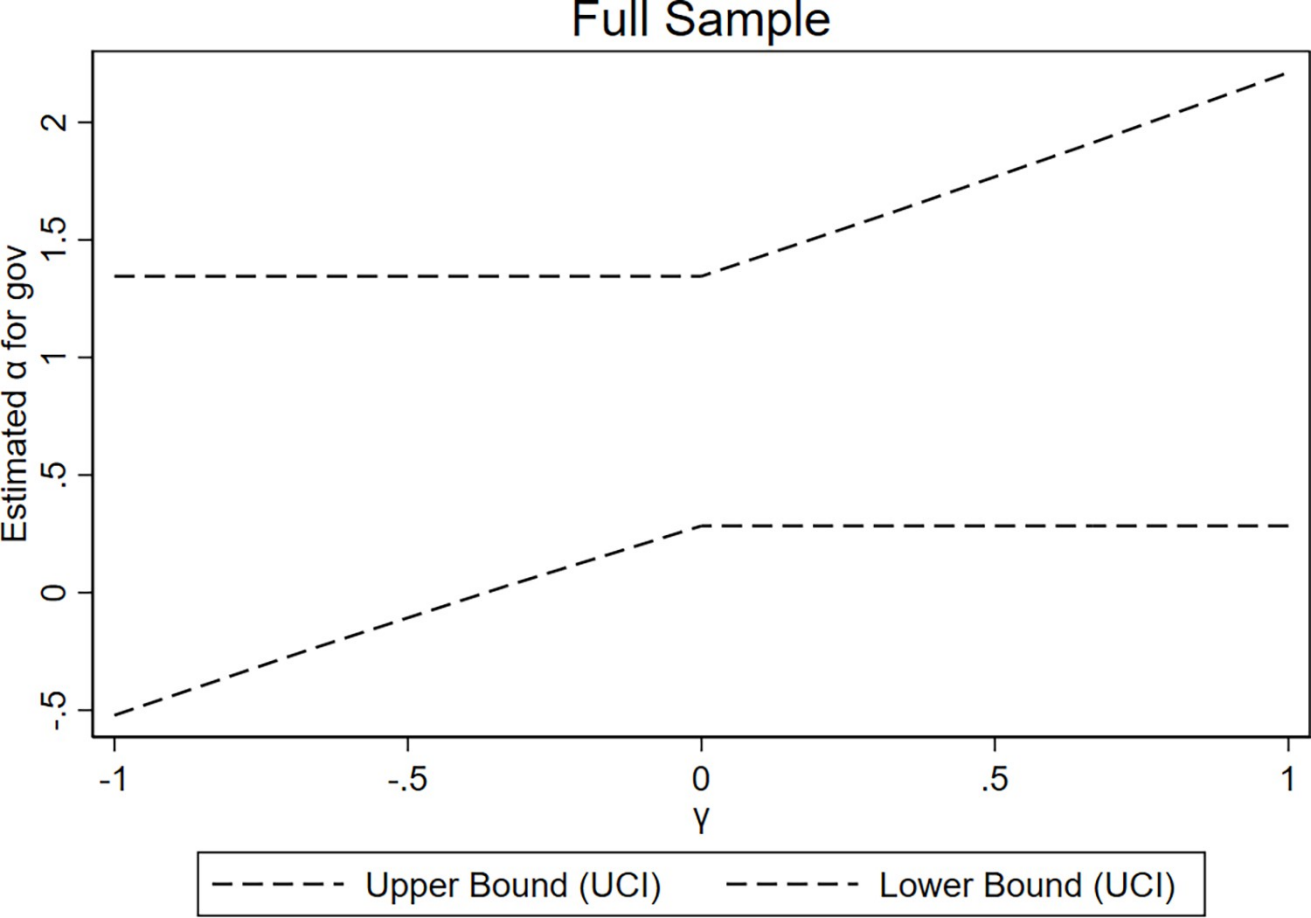
Results of the UCI approach (full sample).

**Fig 4:**
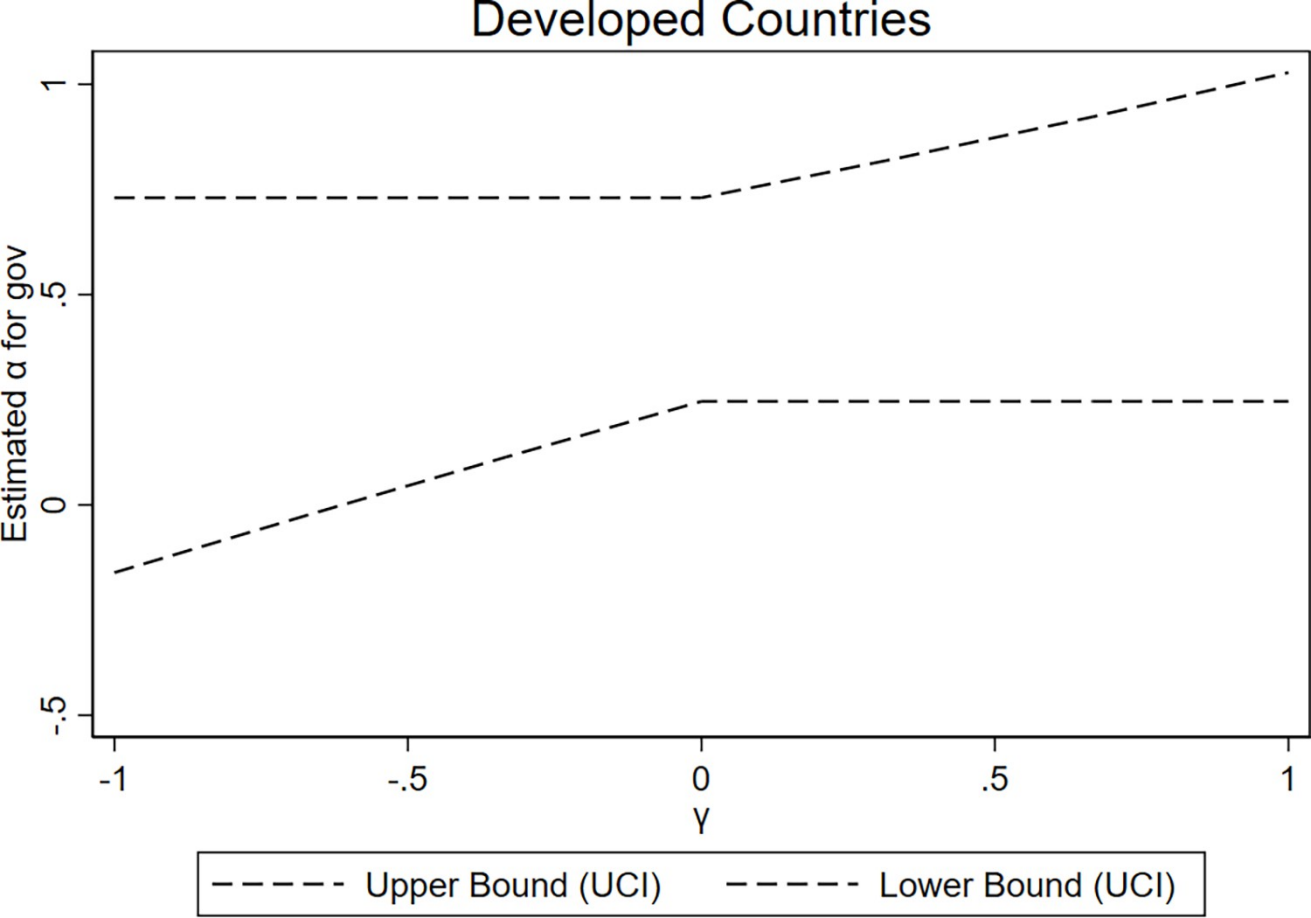
Results of the UCI approach (developed countries).

**Fig 5:**
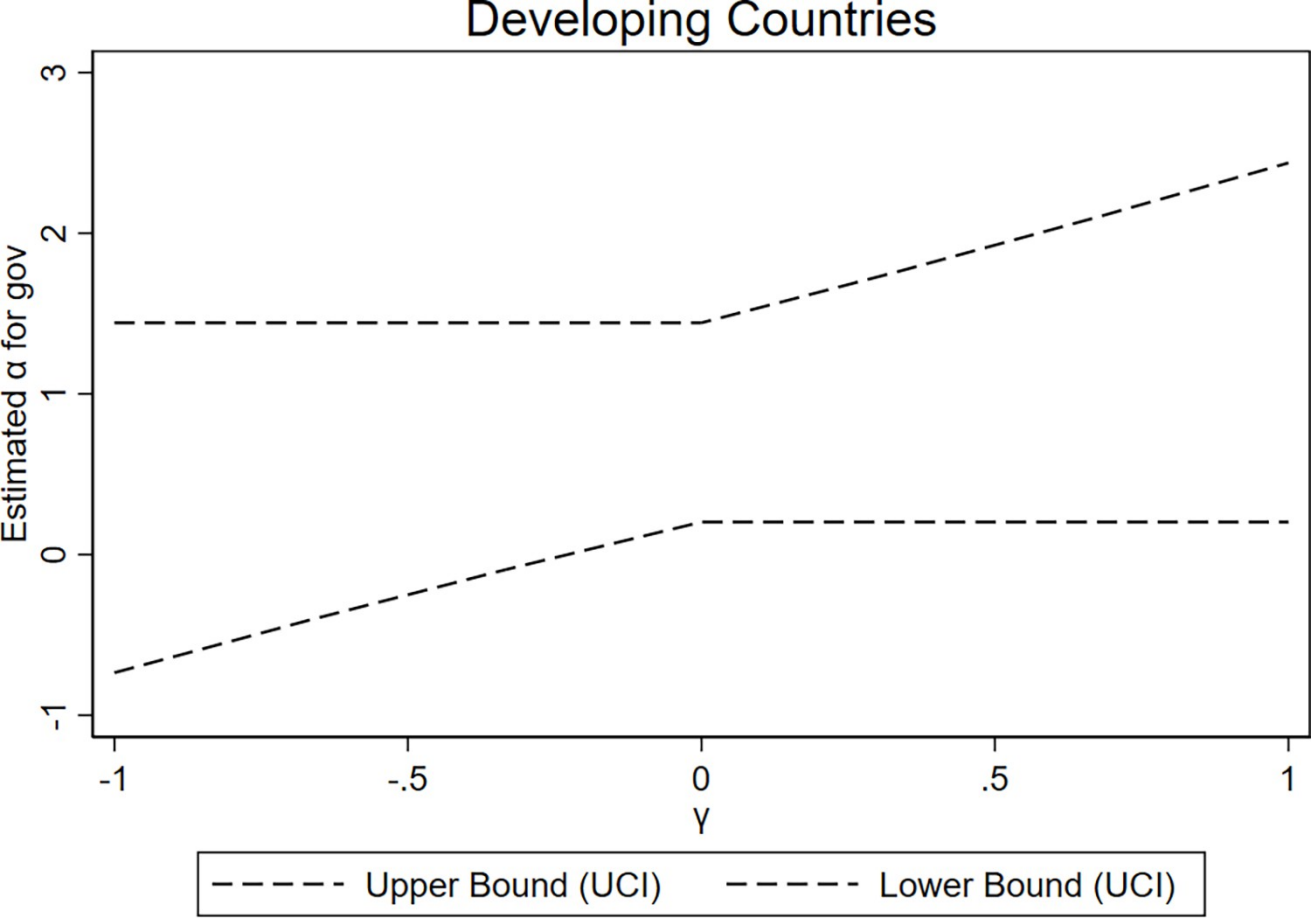
Results of the UCI approach (developing countries).

We set the support set of *γ* to [–1, 1], with a confidence level of 90%, and present the graphical results of the UCI approach (refer to Figs 3–5). In Fig 3, using the entire sample as an example within the support set range, *γ* is approximately -0.37 when the 90% combined upper limit of the confidence interval intersects with the zero limit of the horizontal axis. This result indicates that *α*_1_ is significantly positive as long as *γ* is greater than -0.37. Furthermore, at *γ* approximately -0.37, it accounts for 36% of the total effect of military expenditures (the coefficient of *mil* in Column (1) in Table 8). This result indicates that even when instrumental variables violate the exclusion restriction to a considerable extent, significant positive results for *α*_1_ can still be obtained, thus supporting the twin deficit hypothesis. In developed and developing countries, this value of *γ* is -0.63 and -0.23, accounting for 43% and 25% of the corresponding total effect of military expenditures (the coefficients of *mil* in Columns (2) and (3) in Table 8), respectively, further demonstrating the robustness of the results.

### Robustness test

In this section, we discuss the replacement of variable indicators, incorporation of additional control variables, exclusion of samples potentially exhibiting abnormal capital flows, and averaging data every five years, all aimed at mitigating the potential influence of omitted variables and measurement errors.

First, inspired by previous research [57,58], we take the ratio of the merchandise and service trade balance to GDP as an indicator of the current account deficit for robustness testing. Second, to control the impact of foreign net assets, we sacrifice the regression sample size and includes the foreign net asset variable in the regression. Third, we exclude four countries with international financial centers, namely, the United Kingdom, Germany, Switzerland, and Singapore, as well as OPEC countries, as these may face abnormal capital flows [32]. Fourth, we average all variable data every five years to reduce the impact of economic cycles and measurement errors [14]. Fifth, to control for the impact of fiscal rule variables, we include the four fiscal rule variables (ER, RR, BBR, and DR) in the regressions at the expense of reducing the number of regression samples. The regression results (see Tables 9–12) still exhibit a significantly positive coefficient for the budget deficit, thus supporting the twin deficit hypothesis. It should also be noted that excluding the samples with abnormal capital flows and averaging data every five years significantly reduces the sample size in the regressions, which may lead to a certain degree of weak instrument problem.

**Table 9:** Replacing current account deficit measurement indicator and controlling foreign net assets.

	Full sample	Developed countries	Developing countries	Full sample	Developed countries	Developing countries
	(1)	(2)	(3)	(4)	(5)	(6)
*gov*	0.898***	0.788***	0.927***	0.805***	0.490***	0.814***
	(0.252)	(0.189)	(0.317)	(0.204)	(0.172)	(0.246)
*gdpg*	-0.200***	-0.334**	-0.177***	-0.191***	-0.199**	-0.197***
	(0.061)	(0.169)	(0.064)	(0.061)	(0.101)	(0.063)
*gdpp*	0.028*	-0.062	0.042**	-0.020	-0.064*	0.002
	(0.015)	(0.038)	(0.017)	(0.019)	(0.034)	(0.021)
*tot*	0.036*	-0.027	0.045**	0.004	-0.123	0.016
	(0.019)	(0.089)	(0.019)	(0.020)	(0.087)	(0.020)
*yth*	-0.039	-0.089	-0.069	-0.101***	0.226**	-0.159***
	(0.037)	(0.128)	(0.045)	(0.028)	(0.105)	(0.034)
*old*	0.797***	0.096	0.820***	0.972***	-0.141	0.874***
	(0.115)	(0.155)	(0.182)	(0.119)	(0.189)	(0.176)
*fd*	-0.029***	-0.041***	-0.048**	-0.023***	-0.012*	-0.063***
	(0.008)	(0.008)	(0.021)	(0.008)	(0.007)	(0.019)
*open*	-0.012	0.082***	-0.021	0.005	0.100***	0.004
	(0.016)	(0.022)	(0.018)	(0.013)	(0.025)	(0.015)
*ka*	-0.003	-0.003	-0.002	-0.002	0.006	-0.002
	(0.002)	(0.004)	(0.002)	(0.002)	(0.004)	(0.002)
*errcm*	0.003***	-0.001	0.004***	0.001	0.003***	0.001
	(0.001)	(0.001)	(0.001)	(0.001)	(0.001)	(0.001)
*nfa*				0.029***	0.000	0.031***
				(0.006)	(0.006)	(0.007)
Country FE	Yes	Yes	Yes	Yes	Yes	Yes
Year FE	Yes	Yes	Yes	Yes	Yes	Yes
N	2462	510	1952	2347	443	1904
R^2^	0.064	0.126	0.097	0.071	0.309	0.103
**First-stage regression results**						
*mil*	-1.245***	-3.007***	-1.021***	-1.237***	-3.179***	-1.078***
	(0.173)	(0.425)	(0.176)	(0.161)	(0.434)	(0.162)
K-P rk LM	40.599***	42.109***	25.771***	50.904***	40.502***	36.689***
C-D Wald F	107.671	40.025	67.538	101.923	41.925	71.921
K-P Wald rk F	51.561	50.162	33.814	58.700	53.742	44.060
A-R Wald test F	9.45***	16.81***	6.18**	17.77***	6.28**	12.43***

Note

*, **, and *** denote statistical significance at the 10%, 5%, and 1% levels, respectively. The values in parentheses are cluster-robust standard errors, clustered at the country level.

**Table 10:** Exclusion of samples with abnormal capital flows and averaging data every five years.

	Full sample	Developed countries	Developing countries	Full sample	Developed countries	Developing countries
	(1)	(2)	(3)	(4)	(5)	(6)
*gov*	0.842***	0.638***	0.822***	0.747*	0.551**	1.070
	(0.221)	(0.150)	(0.255)	(0.407)	(0.250)	(0.725)
*gdpg*	-0.173***	-0.214*	-0.174***	-0.124	-0.338	-0.095
	(0.057)	(0.122)	(0.058)	(0.138)	(0.228)	(0.189)
*gdpp*	-0.002	-0.095***	0.019	0.022	-0.020	-0.007
	(0.015)	(0.031)	(0.017)	(0.034)	(0.055)	(0.050)
*tot*	-0.001	-0.187**	0.011	-0.031	-0.012	-0.045
	(0.018)	(0.074)	(0.018)	(0.039)	(0.226)	(0.049)
*yth*	-0.080***	0.117	-0.135***	-0.092**	0.336**	-0.112
	(0.025)	(0.108)	(0.031)	(0.043)	(0.160)	(0.075)
*old*	0.692***	0.081	0.601***	0.649***	-0.294	0.756*
	(0.092)	(0.161)	(0.138)	(0.179)	(0.343)	(0.428)
*fd*	-0.030***	-0.026***	-0.078***	-0.027*	-0.015	-0.048
	(0.008)	(0.008)	(0.019)	(0.014)	(0.013)	(0.043)
*open*	-0.001	0.089***	0.003	0.005	0.111***	-0.013
	(0.011)	(0.023)	(0.012)	(0.020)	(0.030)	(0.038)
*ka*	-0.002	0.008**	-0.002	-0.004	0.005	-0.001
	(0.002)	(0.004)	(0.002)	(0.003)	(0.007)	(0.006)
*errcm*	0.000	0.001	0.000	0.001	0.002	0.002
	(0.001)	(0.001)	(0.001)	(0.001)	(0.002)	(0.002)
Country FE	Yes	Yes	Yes	Yes	Yes	Yes
Year FE	Yes	Yes	Yes	Yes	Yes	Yes
N	2629	428	2201	615	88	383
R^2^	0.077	0.304	0.114	0.112	0.511	-0.026
**First-stage regression results**						
*mil*	-1.263***	-3.290***	-1.116***	-1.10***	-3.878***	-0.694***
	(0.160)	(0.479)	(0.202)	(0.262)	(1.043)	(0.227)
K-P rk LM	48.860***	40.828***	37.608***	13.641***	10.131***	6.413**
C-D Wald F	121.178	30.488	91.766	23.157	12.337	8.585
K-P Wald rk F	62.326	47.209	48.903	17.517	13.832	9.368
A-R Wald test F	12.13***	11.57***	8.66***	3.18*	2.49	2.38

Note

*, **, and *** denote statistical significance at the 10%, 5%, and 1% levels, respectively. The values in parentheses are cluster-robust standard errors, clustered at the country level.

**Table 11:** Controlling for expenditure rules and revenue rules.

	Full sample	Developed countries	Developing countries	Full sample	Developed countries	Developing countries
	(1)	(2)	(3)	(4)	(5)	(6)
*gov*	0.833**	0.465**	0.813*	0.814**	0.503***	0.806*
	(0.323)	(0.186)	(0.474)	(0.326)	(0.168)	(0.473)
*gdpg*	-0.193***	-0.109	-0.197**	-0.188***	-0.093	-0.195**
	(0.070)	(0.099)	(0.081)	(0.070)	(0.098)	(0.080)
*gdpp*	0.025	-0.059	0.057**	0.023	-0.080**	0.056*
	(0.024)	(0.038)	(0.028)	(0.024)	(0.033)	(0.029)
*tot*	-0.003	-0.153*	0.002	-0.001	-0.117	0.002
	(0.029)	(0.078)	(0.029)	(0.029)	(0.081)	(0.029)
*yth*	-0.076**	0.161	-0.121***	-0.066*	0.263**	-0.118***
	(0.038)	(0.102)	(0.039)	(0.038)	(0.106)	(0.041)
*old*	0.479***	0.133	0.428**	0.494***	0.058	0.447**
	(0.105)	(0.161)	(0.217)	(0.112)	(0.148)	(0.211)
*fd*	-0.021**	-0.020***	-0.078***	-0.020**	-0.019***	-0.078***
	(0.008)	(0.007)	(0.025)	(0.008)	(0.007)	(0.026)
*open*	0.032**	0.059***	0.036*	0.035**	0.062***	0.037*
	(0.014)	(0.018)	(0.020)	(0.014)	(0.018)	(0.020)
*ka*	-0.007***	0.006	-0.007***	-0.007***	0.004	-0.007***
	(0.002)	(0.004)	(0.002)	(0.002)	(0.004)	(0.002)
*errcm*	0.000	-0.000	0.000	0.000	-0.001	0.000
	(0.001)	(0.001)	(0.001)	(0.001)	(0.001)	(0.001)
*ER*	0.007	0.012**	0.003			
	(0.005)	(0.005)	(0.007)			
*RR*				-0.015**	-0.021***	-0.004
				(0.006)	(0.005)	(0.011)
Country FE	Yes	Yes	Yes	Yes	Yes	Yes
Year FE	Yes	Yes	Yes	Yes	Yes	Yes
N	1742	486	1256	1742	486	1256
R^2^	0.058	0.319	0.110	0.065	0.311	0.111
**First-stage regression results**						
*mil*	-1.159***	-2.906***	-0.850***	-1.167***	-3.123***	-0.853***
	(0.192)	(0.463)	(0.203)	(0.191)	(0.454)	(0.200)
K-P rk LM	27.012***	35.154***	12.599***	27.155***	39.120***	13.059***
C-D Wald F	72.809	34.055	35.793	73.749	39.313	36.398
K-P Wald rk F	36.295	39.383	17.469	37.167	47.407	18.270
A-R Wald test F	5.32**	4.76**	2.24	5.04**	6.86***	2.24

Note

*, **, and *** denote statistical significance at the 10%, 5%, and 1% levels, respectively. The values in parentheses are cluster-robust standard errors, clustered at the country level.

**Table 12:** Controlling for balanced budget rules and debt rules.

	Full sample	Developed countries	Developing countries	Full sample	Developed countries	Developing countries
	(1)	(2)	(3)	(4)	(5)	(6)
*gov*	0.897***	0.527***	0.868*	0.868***	0.523***	0.877*
	(0.348)	(0.167)	(0.525)	(0.331)	(0.169)	(0.500)
*gdpg*	-0.203***	-0.110	-0.204**	-0.201***	-0.106	-0.209**
	(0.073)	(0.100)	(0.085)	(0.072)	(0.101)	(0.085)
*gdpp*	0.022	-0.083**	0.055*	0.024	-0.083**	0.055*
	(0.024)	(0.032)	(0.029)	(0.024)	(0.033)	(0.028)
*tot*	-0.001	-0.128	0.002	0.003	-0.122	0.005
	(0.029)	(0.081)	(0.030)	(0.029)	(0.082)	(0.029)
*yth*	-0.065*	0.202*	-0.112***	-0.063*	0.197*	-0.105***
	(0.037)	(0.110)	(0.040)	(0.038)	(0.110)	(0.041)
*old*	0.462***	0.078	0.429**	0.450***	0.079	0.389*
	(0.123)	(0.149)	(0.218)	(0.119)	(0.149)	(0.225)
*fd*	-0.021***	-0.021***	-0.075***	-0.023***	-0.021***	-0.077***
	(0.008)	(0.007)	(0.027)	(0.008)	(0.007)	(0.025)
*open*	0.036**	0.064***	0.037*	0.034**	0.064***	0.035*
	(0.014)	(0.018)	(0.020)	(0.014)	(0.018)	(0.020)
*ka*	-0.006***	0.006	-0.006***	-0.006***	0.006*	-0.006***
	(0.002)	(0.004)	(0.002)	(0.002)	(0.004)	(0.002)
*errcm*	0.000	-0.000	0.000	0.000	-0.000	-0.000
	(0.001)	(0.001)	(0.001)	(0.001)	(0.001)	(0.001)
*BBR*	-0.011*	0.001	-0.005			
	(0.006)	(0.007)	(0.007)			
*DR*				-0.013***	-0.002	-0.010
				(0.005)	(0.006)	(0.007)
Country FE	Yes	Yes	Yes	Yes	Yes	Yes
Year FE	Yes	Yes	Yes	Yes	Yes	Yes
N	1742	486	1256	1742	486	1256
R^2^	0.037	0.292	0.096	0.048	0.293	0.095
**First-stage regression results**						
*mil*	-1.085***	-3.143***	-0.775***	-1.136***	-3.105***	-0.806***
	(0.192)	(0.433)	(0.202)	(0.192)	(0.450)	(0.201)
K-P rk LM	24.102***	41.329***	10.937***	26.177***	38.196***	11.888***
C-D Wald F	62.937	41.031	28.845	69.556	38.735	31.852
K-P Wald rk F	32.092	52.730	14.692	35.157	47.712	16.135
A-R Wald test F	5.28**	7.81***	2.07	5.47**	7.30***	2.33

Note

*, **, and *** denote statistical significance at the 10%, 5%, and 1% levels, respectively. The values in parentheses are cluster-robust standard errors, clustered at the country level.

## Conclusion

Does the twin deficit hypothesis hold? Although many cross-country empirical studies indicate a significant positive correlation between budget and current account deficits, they often face endogeneity issues due to reverse causality, thereby making it challenging to establish the validity of the hypothesis. We used cross-country panel data with military expenditure as an instrumental variable for budget deficits to test the validity of the twin deficit hypothesis. Our findings reveal that in both developed and developing countries, an increase in budget deficits leads to a widening of current account deficits, thus confirming the validity of the twin deficit hypothesis. The hypothesis remains valid after considering various factors that may affect the exogeneity of military expenditures and instrumental variable approximation exogeneity and multiple robustness tests.

This study provides evidence supporting the use of fiscal policies to address imbalances in the current account. For global current account imbalances, countries with significant current account deficits should appropriately reduce government spending in certain situations to decrease the deficit, while those with significant surpluses can increase government spending to reduce this. For individual countries, if a country implements an expansionary fiscal policy, the fiscal policy should ensure continuity and moderate strength to avoid a large budget deficit and to prevent large fluctuations and deficits in the current account.

## Supporting information

S1 Table The relevant information about countries and years in the sample. (DOCX)

S1 Data Data and code. (XLS)

S2 Data Data and code. (XLS)
