# Supplementary material for: Do budget deficits cause current account deficits? a re-evaluation utilizing military expenditures as an instrumental variable
Source: PLoS One. 2024 Oct 25;19(10):e0311664. doi: 10.1371/journal.pone.0311664 (PMC11508486; doi:10.1371/journal.pone.0311664)
Supplement: S1 Table — (DOCX) [file pone.0311664.s001.docx]

**S1 Table. The relevant information about countries and years in the sample.**

| **Developed countries** | | | | | **Developing countries** | | | | | | | | | |
| --- | --- | --- | --- | --- | --- | --- | --- | --- | --- | --- | --- | --- | --- | --- |
| Num | ISO code | Countries | Start-stop year | | Num | ISO code | Countries | Start-stop year | | Num | ISO code | Countries | Start-stop year | |
| 1 | AUS | Australia | 1991 | 2018 | 1 | AGO | Angola | 2000 | 2018 | 55 | LBN | Lebanon | 1990 | 2017 |
| 2 | AUT | Austria | 2001 | 2018 | 2 | ALB | Albania | 1995 | 2018 | 56 | LBR | Liberia | 2007 | 2018 |
| 3 | BEL | Belgium | 2001 | 2018 | 3 | ARE | UAE | 2001 | 2014 | 57 | LKA | Sri Lanka | 2004 | 2018 |
| 4 | CAN | Canada | 1992 | 2008 | 4 | ARG | Argentina | 1993 | 2017 | 58 | LSO | Lesotho | 2007 | 2018 |
| 5 | CHE | Switzerland | 1996 | 2016 | 5 | ARM | Armenia | 2002 | 2018 | 59 | LTU | Lithuania | 2010 | 2018 |
| 6 | CYP | Cyprus | 2001 | 2018 | 6 | AZE | Azerbaijan Rep. of | 1996 | 2017 | 60 | LVA | Latvia | 2010 | 2018 |
| 7 | DEU | Germany | 2001 | 2018 | 7 | BDI | Burundi | 1990 | 2000 | 61 | MAR | Morocco | 1990 | 2018 |
| 8 | DNK | Denmark | 1990 | 2018 | 8 | BEN | Benin | 1999 | 2018 | 62 | MDA | Moldova | 1996 | 2018 |
| 9 | ESP | Spain | 2001 | 2018 | 9 | BFA | Faso | 1990 | 2018 | 63 | MDG | Madagascar | 1990 | 2018 |
| 10 | FIN | Finland | 2001 | 2018 | 10 | BGD | Bangladesh | 1990 | 2018 | 64 | MEX | Mexico | 1990 | 2018 |
| 11 | FRA | France | 2001 | 2018 | 11 | BGR | Bulgaria | 1994 | 2018 | 65 | MKD | Macedonia FYR | 1997 | 2018 |
| 12 | GBR | United Kingdom | 2001 | 2018 | 12 | BHR | Bahrain Kingdom of | 1990 | 2015 | 66 | MLI | Mali | 1990 | 2018 |
| 13 | GRC | Greece | 2001 | 2018 | 13 | BIH | Bosnia & Herzegovina | 2002 | 2018 | 67 | MLT | Malta | 2005 | 2018 |
| 14 | IRL | Ireland | 2001 | 2018 | 14 | BLR | Belarus | 1998 | 2018 | 68 | MNG | Mongolia | 1995 | 2016 |
| 15 | ISR | Israel | 2001 | 2018 | 15 | BLZ | Belize | 1990 | 2018 | 69 | MOZ | Mozambique | 1997 | 2018 |
| 16 | ITA | Italy | 2001 | 2018 | 16 | BOL | Bolivia | 1990 | 2018 | 70 | MRT | Mauritania | 2005 | 2018 |
| 17 | KOR | Korea | 1995 | 2018 | 17 | BRA | Brazil | 1998 | 2018 | 71 | MWI | Malawi | 1990 | 2016 |
| 18 | NLD | Netherlands | 2001 | 2018 | 18 | BWA | Botswana | 1990 | 2018 | 72 | MYS | Malaysia | 1995 | 2018 |
| 19 | NOR | Norway | 1990 | 2018 | 19 | CAF | Central African Rep. | 2002 | 2018 | 73 | NAM | Namibia | 1994 | 2018 |
| 20 | NZL | New Zealand | 1990 | 2018 | 20 | CHL | Chile | 1990 | 2018 | 74 | NER | Niger | 1995 | 2018 |
| 21 | PRT | Portugal | 2001 | 2018 | 21 | CHN | China, PR | 1997 | 2018 | 75 | NGA | Nigeria | 2000 | 2018 |
| 22 | SGP | Singapore | 1994 | 2018 | 22 | CIV | Cote D"Ivoire | 2003 | 2018 | 76 | NIC | Nicaragua | 1994 | 2018 |
| 23 | SWE | Sweden | 1990 | 2018 | 23 | CMR | Cameroon | 1990 | 2018 | 77 | NPL | Nepal | 2005 | 2018 |
| 24 | USA | United States | 1990 | 2018 | 24 | COG | Congo Rep. of | 2001 | 2018 | 78 | OMN | Oman | 1990 | 2018 |
|  |  |  |  |  | 25 | COL | Colombia | 1990 | 2018 | 79 | PAK | Pakistan | 1990 | 2018 |
|  |  |  |  |  | 26 | CPV | Verde | 1996 | 2018 | 80 | PER | Peru | 1990 | 2018 |
|  |  |  |  |  | 27 | CZE | Czech Rep. | 1996 | 2018 | 81 | PHL | Philippines | 1990 | 2018 |
|  |  |  |  |  | 28 | DOM | Dominican Republic | 1992 | 2018 | 82 | POL | Poland | 1995 | 2018 |
|  |  |  |  |  | 29 | DZA | Algeria | 1990 | 2003 | 83 | PRY | Paraguay | 1998 | 2018 |
|  |  |  |  |  | 30 | ECU | Ecuador | 1990 | 2018 | 84 | QAT | Qatar | 2002 | 2010 |
|  |  |  |  |  | 31 | EGY | Egypt | 2002 | 2010 | 85 | RUS | Russia | 2001 | 2018 |
|  |  |  |  |  | 32 | EST | Estonia | 2004 | 2018 | 86 | RWA | Rwanda | 1990 | 2018 |
|  |  |  |  |  | 33 | GAB | Gabon | 2000 | 2018 | 87 | SAU | Saudi Arabia | 2016 | 2017 |
|  |  |  |  |  | 34 | GEO | Georgia | 1996 | 2018 | 88 | SDN | Sudan | 2015 | 2018 |
|  |  |  |  |  | 35 | GHA | Ghana | 1990 | 2018 | 89 | SEN | Senegal | 1990 | 2018 |
|  |  |  |  |  | 36 | GIN | Guinea | 2015 | 2018 | 90 | SLV | El Salvador | 1990 | 2018 |
|  |  |  |  |  | 37 | GMB | Gambia | 2000 | 2007 | 91 | SVK | Slovak Republic | 2006 | 2018 |
|  |  |  |  |  | 38 | GNB | Guinea-Bissau | 2000 | 2017 | 92 | SVN | Slovenia | 2004 | 2018 |
|  |  |  |  |  | 39 | GNQ | Equatorial Guinea | 2007 | 2018 | 93 | SWZ | Swaziland | 1990 | 2015 |
|  |  |  |  |  | 40 | GTM | Guatemala | 1990 | 2018 | 94 | SYC | Seychelles | 1996 | 2018 |
|  |  |  |  |  | 41 | GUY | Guyana | 2000 | 2018 | 95 | TGO | Togo | 1990 | 2018 |
|  |  |  |  |  | 42 | HND | Honduras | 2000 | 2018 | 96 | THA | Thailand | 2000 | 2018 |
|  |  |  |  |  | 43 | HRV | Croatia | 2002 | 2018 | 97 | TJK | Tajikistan | 1998 | 2015 |
|  |  |  |  |  | 44 | HUN | Hungary | 1995 | 2018 | 98 | TUN | Tunisia | 1997 | 2018 |
|  |  |  |  |  | 45 | IDN | Indonesia | 1990 | 2018 | 99 | TUR | Turkey | 1990 | 2018 |
|  |  |  |  |  | 46 | IND | India | 1997 | 2018 | 100 | TZA | Tanzania | 1990 | 2017 |
|  |  |  |  |  | 47 | IRN | Iran | 1990 | 2016 | 101 | UGA | Uganda | 1992 | 2018 |
|  |  |  |  |  | 48 | JAM | Jamaica | 1990 | 2018 | 102 | UKR | Ukraine | 1996 | 2013 |
|  |  |  |  |  | 49 | JOR | Jordan | 1990 | 2018 | 103 | URY | Uruguay | 2012 | 2018 |
|  |  |  |  |  | 50 | KAZ | Kazakhstan | 1996 | 2018 | 104 | VEN | Venezuela | 1998 | 2011 |
|  |  |  |  |  | 51 | KEN | Kenya | 1990 | 2018 | 105 | VNM | Vietnam | 1992 | 2011 |
|  |  |  |  |  | 52 | KGZ | Kyrgyz Rep. | 1997 | 2018 | 106 | ZAF | South Africa | 1995 | 2018 |
|  |  |  |  |  | 53 | KHM | Cambodia | 1997 | 2015 | 107 | ZMB | Zambia | 1994 | 2016 |
|  |  |  |  |  | 54 | LAO | Lao Dem. Rep. | 2000 | 2010 |  |  |  |  |  |
